# Role of bisphenol A in the aberrant activation of ionotropic glutamate transporters in the cerebral cortex and altered behavioral responses in C57BL/6J mice

**DOI:** 10.3389/ftox.2025.1680589

**Published:** 2026-01-12

**Authors:** Jasim Khan, Mohammad Waseem, Kajal Kamble, Suhel Parvez, Basu Dev Banerjee, Sarika Gupta, Sheikh Raisuddin

**Affiliations:** 1 Molecular Toxicology Laboratory, Department of Medical Elementology and Toxicology, School of Chemical and Life Sciences, Jamia Hamdard, New Delhi, India; 2 Neurotoxicology Laboratory, Department of Medical Elementology and Toxicology, School of Chemical and Life Sciences, Jamia Hamdard, New Delhi, India; 3 Molecular Sciences Laboratory, National Institute of Immunology, New Delhi, India

**Keywords:** xenobiotics, glutamate transporters, excitotoxicity, oxidative stress, memory impairment

## Abstract

Bisphenol A (BPA) is a widely used endocrine-disrupting chemical that is used to manufacture epoxy resins and polycarbonate plastics. Dietary intake is considered the primary source of human exposure through leaching into food and beverages in contact with storage containers. BPA alters brain function through a wide variety of mechanisms, including oxidative stress, endocrine disruption, developmental toxicity, inflammation, epigenetic modifications, and altered neurotransmission systems. Long-term exposure to even small concentrations of BPA has been associated with neurotoxicity. Mechanistic underpinning of neurotransmitters and free radical-mediated neurotoxicity of BPA is linked with Glutamate (Glu), which plays a vital role in normal brain functioning. Excitatory amino acid transporters (EAATs) play a crucial role in maintaining normal levels of Glu in the synaptic cleft, and EAAT dysfunction leads to excitotoxicity. We studied the effect of oral BPA exposure (40 μg/kg and 400 μg/kg doses) for 60 days in male mice. BPA exposure caused altered spatial learning and deteriorated sensorimotor coordination in exposed animals. These findings were supported by a decrease in acetylcholinesterase (AChE) activity and an increase in monoamine oxidase (MAO), coupled with nitrosative and oxidative overload in the cerebral cortex. A significant upregulation in expression of EAATs and xCT was observed in BPA-treated animals compared to controls at mRNA and protein levels in the cerebral cortex. BPA also caused histopathological changes in the cortical and hippocampal regions of exposed mice. Results of our study prove that even low-dose BPA exposure caused neurotoxicity and altered the expression of Glu-transporters with putative behavioral changes.

## Graphical Abstract

**Figure:**
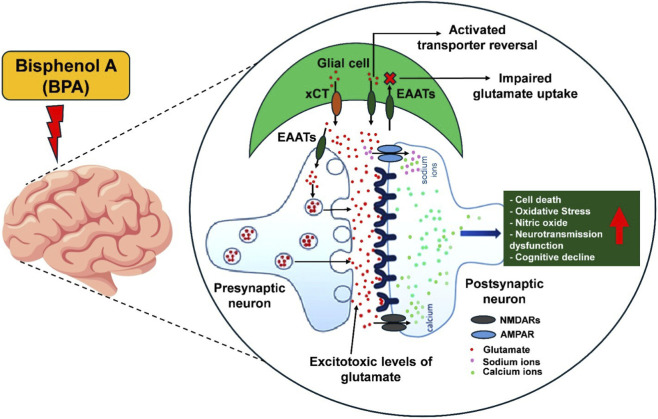

## Highlights

- BPA exposure caused learning and motor defects.
- BPA exposure increased glutamate transporter and antiporter expression.
- BPA exposure dysregulated acetylcholinesterase and monoamine oxidase activity.
- BPA induces lipid peroxidation *via* oxidative stress.
- BPA exposure caused histopathological changes in the hippocampus and cortical tissue.

## 1 Introduction

Bisphenol A (BPA) is a high-production industrial chemical that is also a potent endocrine-disrupting chemical (EDC), with global demand projected to increase to 10.6 million metric tons by 2022 (Lehmler et al., 2018). BPA exposure in humans occurs ubiquitously through food, contaminated water, air, and dust (Kang et al., 2006; Wang et al., 2020; González-Rubio et al., 2020). Inefficient wastewater treatment allows BPA to be released with effluents and get mixed with drinking water (Tursi et al., 2018). High concentrations of BPA have been reported in human tissues and fluids across all age groups (vom Saal and Welshons, 2014; Lehmler et al., 2018). Widespread exposure to consumer products, including plastic utensils, dental sealants, thermal papers, and medical devices, contributes significantly to its exposure in humans (Schönfelder et al., 2002; Ribeiro et al., 2017). The toxic effects of BPA have been investigated both at the molecular and cellular levels (Richter et al., 2007; Wang et al., 2019b; Anjum et al., 2011; Khan et al., 2019. Due to its strong lipophilicity and small chemical size, BPA has been reported to cross the blood-brain barrier (BBB) (Sun et al., 2002; Negri-Cesi, 2015). This makes BPA a chemical entity of serious concern, as it can hamper the neurochemical homeostasis and cause behavioral abnormalities, potentially after crossing the BBB (Ma et al., 2021). BPA has been implicated in some neurological disorders, such as amyotrophic lateral sclerosis (ALS) (Preciados et al., 2016), autism spectrum disorders (Stein et al., 2015), multiple sclerosis (Rogers et al., 2017), attention deficit hyperactivity disorder (ADHD) (Tavakkoli et al., 2020), schizophrenia (Brown, 2009), and epilepsy (Crino et al., 2002; Tavakkoli et al., 2020). Also, its demyelinating effect and axonal damage establish BPA as a potential neurotoxic chemical of concern (Khan et al., 2018; Khan et al., 2019). BPA has been reported to affect hippocampal functionality with concomitant memory and behavior deficits (Suresh et al., 2022; Luo et al., 2023).

Glutamate (Glu) is the major excitatory neurotransmitter of the central nervous system (CNS), and the peripheral nervous system (PNS) plays a pivotal role in maintaining complex cellular communication networks within brain cells, including neuronal and glial cells (Carozzi et al., 2008; Köles et al., 2016). Aberrant Glu activity could affect physiological functions and interactions of brain cells, leading to brain pathologic conditions like schizophrenia (O’Donovan et al., 2017; Olasehinde et al., 2019). Optimal Glu concentration and homeostasis are maintained by Glu transporters, namely, excitatory amino acid transporter 1-5 (EAAT1, EAAT2, EAAT3, EAAT4, and EAAT5) (Danbolt, 2001; Zhou and Danbolt, 2013). Out of these transporters, EAAT1 and EAAT2 are mainly localized on glial cells, whereas EAAT3 is primarily localized on neurons (Danbolt, 2001; Zhou and Danbolt, 2013). Expressions of EAAT4 and EAAT5 are confined to the cerebellum and retina, respectively (Abousaab et al., 2016; Martinez-Lozada et al., 2016). Glu transporters have their rodent homologues as Glu-aspartate transporter (GLAST) for EAAT1, Glu transporter-1 (GLT-1) for EAAT2, and excitatory amino acid transporter 1 (EAAC1) for EAAT3, respectively. EAAT4 and EAAT5 annotations remained the same, both in rodents and humans. These transporters are responsible for the removal of Glu from the synaptic cleft, both in neurons and glial cells, to maintain the lower concentrations of extracellular Glu, which resultantly protects from excitotoxicity-induced neuronal cell damage (Danbolt, 2001; Zhou and Danbolt, 2013). EAAT2 is the most abundant and active transporter for limiting Glu overload across the membrane (Zhou and Danbolt, 2013; Abousaab et al., 2016). On the other hand, xCT (SLC7A11) is another type of Glu transporter/antiporter responsible for the influx of cystine and efflux of Glu in a controlled 1:1 ratio across the membrane (Koppula et al., 2018). Increased activity of xCT transporters could lead to an increase in extracellular Glu levels to the extent of excitotoxicity (Nelson-Mora et al., 2018). Furthermore, knock-out mice of xCT transporters showed a 50% reduction in the extracellular Glu, highlighting its utility to maintain the Glu homeostasis across the membrane in the mouse model of epilepsy and Parkinson’s disease (De Bundel et al., 2011; Massie et al., 2011). It is reported that there is an increased extracellular Glu and downregulation of both α-amino3-hydroxy-5-methyl-4-isoxazolepropionic acid (AMPA) and N-methyl-D-aspartate (NMDA) Glu receptors when xCT is overexpressed. These changes are also correlated with memory-related alterations (Ramos-Chávez et al., 2015; Nelson-Mora et al., 2018). Also, Glu is involved in normal brain functioning and expression of these transporters is the highest in the cerebral cortex, which made us focus towards analyzing this area of brain which controls diverse human responses including, decision-making, language, memory, reasoning, thought, learning, emotion and intelligence (Fuster, 2009; O’Donovan et al., 2017; Olasehinde et al., 2019).

BPA has been reported to be neurotoxic by negatively affecting the Glu axis in the brain (Wang et al., 2019a). Glu receptor-mediated neurotoxic responses are well reported in relation to BPA exposure, but no relationship between Glu transporters/antiporter activity and related neurobehavioral outcomes has been studied yet (Wang et al., 2019a). It is worth noting that BPA disrupts Glu regulation in both the CNS and PNS (Costa and Cairrao, 2024). In CNS, it acts through altering NMDA and AMPA-mediated synaptic functions, and the PNS, it increases the excitability in dorsal root ganglia and nociception in exposed animals (Soriano et al., 2019). These findings show that studying the effects of BPA on Glu transporters due to their potential role in both CNS and PNS-mediated cognitive and sensory responses, will uncover novel mechanisms of its neurotoxicity. In this study, we focused on the effects of BPA exposure on the Glu transporters/antiporters in the cerebral cortex of male C57BL/6J mice. The overall aim of this study is to elucidate the role of Glu transporter/antiporter in BPA-induced alterations of neurochemical and associated neurobehavioral changes.

## 2 Materials and methods

### 2.1 Chemicals

All chemicals used in this study were purchased from Sigma-Aldrich Company (St. Louis, MO, United States) unless otherwise indicated. Antibodies were purchased from Abcam, UK, Santa Cruz Biotechnology, CA, United States, and Cell Signaling Technology, MA, United States (Details given in Supplementary Material, Supplementary Table S1).

### 2.2 Animals and treatments

**FIGURE 1:**
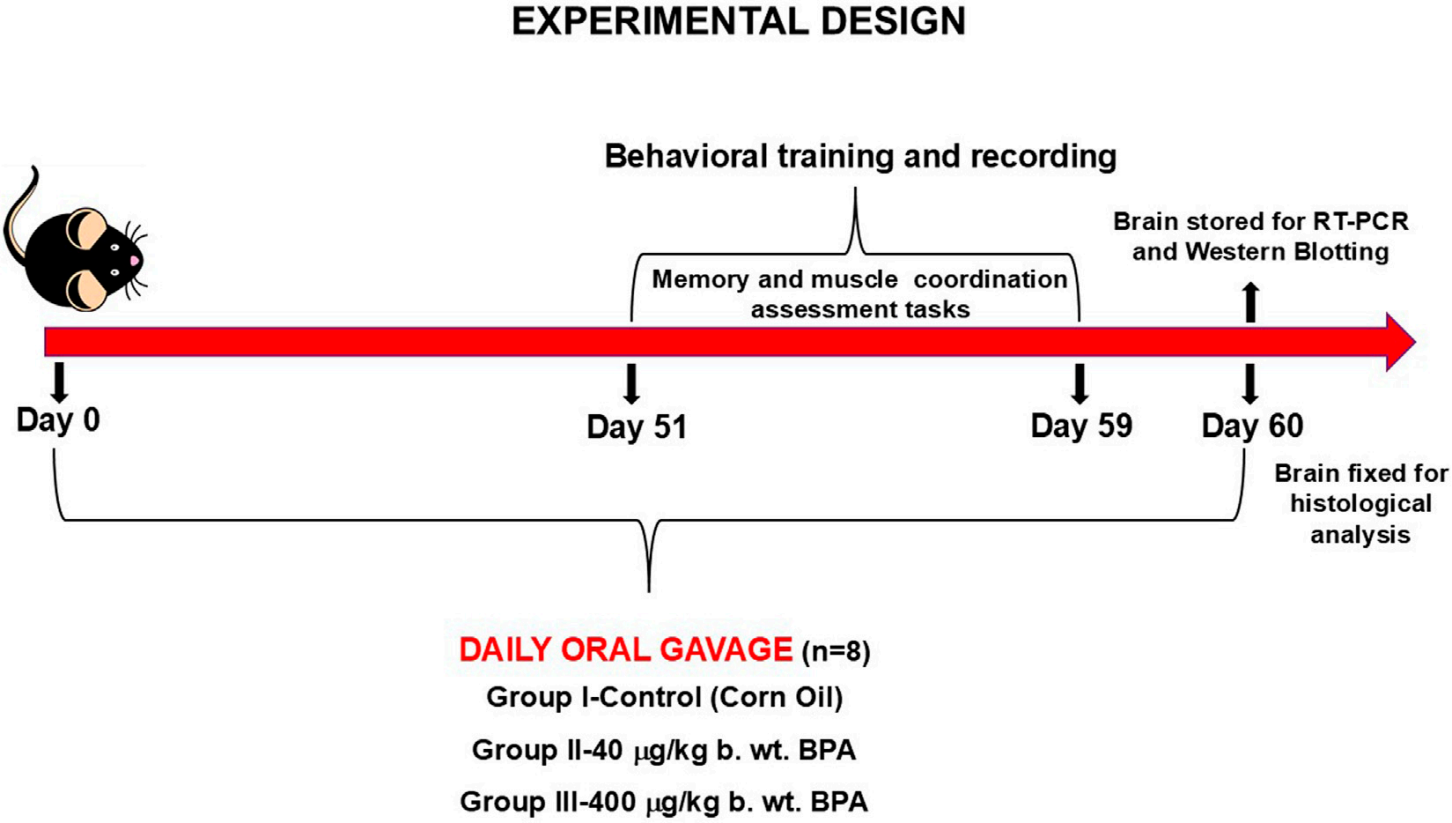
Experimental design of the study.

Aged-matched, eight-week-old C57BL/6J male mice procured from the Central Animal House Facility of Jamia Hamdard (New Delhi, India) were housed at the room temperature of 23 °C ± 2 °C with a 12 h dark/light cycle, and food and water *ad libitum*. To eliminate the effects of the hormone cycle in females, only male mice were included in this study. Animals were bred and maintained as per the guidelines of the Committee for the Purpose of Control and Supervision of Experiments on Animals (CPCSEA). Protocols for animal usage were approved by the Institutional Animal Ethics Committee (Project # 1197). All the animals were randomly selected before grouping and were divided into the three groups (*n* = 8). Doses of BPA were selected based on previously published reports showing neurobehavioral effects *in vivo* (Xu et al., 2011; Kimura et al., 2016). Three groups were: control (corn oil), BPA treatment low dose (40 μg/kg b. wt.), and BPA treatment high dose (400 μg/kg b. wt.) groups. Corn oil was used as a vehicle to suspend BPA. Animals were gavaged daily between 10 a.m. and 11 a.m. Dosing in animals began at 8 weeks of age, and that day was considered day 0; the study was terminated at day 60. The whole intact brains were dissected out, and with a randomized approach, five of them were snap-frozen in liquid nitrogen and then stored at −80 °C for molecular biology analysis. Three whole brains were isolated after PBS perfusion and later fixation in 4% paraformaldehyde for histological analysis. Biochemical parameters were measured on the same day. Study design is graphically presented in Figure 1.

### 2.3 Behavior study

Each mouse received three behavioral training sessions for 3 days, followed by a probe trial on the fourth day. As per the animal behavior assessment guidelines (Rodriguiz and Wetsel, 2006), all animals were brought to the experimental room 30 min before the behavioral recordings to acclimate them to the surroundings. All the animal behavior experiments were performed by the experimenter, blinded to the treatment details within the groups.

#### 2.3.1 Morris water maze test (MWM)

The MWM is a widely accepted assay to assess spatial memory deficit caused by toxicants in neurodegenerative research (Li et al., 2020). In the current study, the MWM task was performed to evaluate sub-chronic BPA-induced behavioral impairment in mice. MWM consisted of a large circular tank of 90 × 45 cm dimensions (diameter × height). Water was filled up to a mark of 30 cm in height in the tank, which was kept at 28 °C ± 1 °C. Using video-tracking software (ANY-maze; Stoelting Co., Wood Dale, IL, United States), four different but equal area quadrants were marked, and a transparent plexiglass circular platform was placed in the center of one of the quadrants. The height of the circular platform was kept 1 cm below the water level. Visual cues were placed to help the mouse recall the position of the platform during the training trials. The position of the platform was kept unchanged for training days, with triangular shaped stickers on the tank wall as a visual cue. Each mouse underwent training in the MWM thrice consecutively for 3 days (3 days of training and a fourth day probe trial with unchanged visual cues) with an inter-trial gap of 5 min. Mice were gently placed in different quadrants in the water-filled pool facing toward the tank wall and left for 90 s to locate the platform, and allowed to stay on the platform for the next 30 s to memorize the safe platform. If a mouse failed to reach the platform within 90 s, it was manually and gently guided to reach the platform and allowed to stay there for the next 30s. The circular platform was removed in the probe trial, and mice were allowed 90 s to look for the hidden platform in the target quadrant. Escape latency and mean swimming speed were measured. Escape latency was calculated from the recordings of four consecutive days (3 days of training and a fourth day probe trial).

#### 2.3.2 Novel object recognition test

To study the effect of BPA on recognition memory, a rectangular polycarbonate box (40 × 23 × 15 cm L × W × H dimensions) was utilized to habituate each mouse (10 min; session on the first day and two 5 min sessions on the second, with a 10 min interval between each session) for 2 consecutive days (Lueptow, 2017). On the 3rd day, mice (n = 8) were exposed to two identical objects (same color) placed at opposite ends of the arena for 10 min. 24 h later, on the final probe trial, mice were allowed to explore one copy of the previously presented object (familiar) and a new object (novel) for 5 min. Overhead-mounted video camera recorded each session, and each recording was analyzed using a stopwatch. Exploration was defined as pointing the head towards an object at 1 cm from the object with an extended neck, and moving the vibrissae. Turning around and sitting on the objects were not counted as exploratory behaviors. The exploration time represents the percentage of time the mice spent exploring the object (familiar or novel) compared to the total exploration time (familiar + novel). Novel object discrimination index was calculated by using the formula: time spent with the novel object/the total object exploration time ×100.

#### 2.3.3 Grip strength (string test)

Grip strength was analyzed by utilizing the string test (Moran et al., 1995). Briefly, the apparatus has a string of 50 cm in length connected between two vertical poles and is elevated. Mice were made to hang on the horizontal string and the grip strength to hold the string was analyzed according to the following score scale: 0—fall off; 1— hangs onto string by two forepaws; 2—as for 1, but attempts to climb on string; 3—hangs onto string by two forepaws plus one or both hind limbs; 4—hangs onto string by forepaws plus tail wrapped around string, and 5— escapes. The highest reading of three successive trials was taken for each animal (*n* = 8).

#### 2.3.4 Rotarod test

To assess sensorimotor coordination, mice were evaluated in the rotarod task before being euthanized (Yasuda et al., 2005). Four trials were conducted using the rotarod apparatus from Orchid Scientific (Nashik, India).

Four trials were conducted with each animal with an interval of 5 min, at the maximum trial length of 180 s, and the speed set at 10 cycles. The instrument was equipped with an infrared-assisted auto-cut for the rotating rod when a mouse falls off the rotating shaft. Data was presented as a mean of time on the rotating bar over the four test trials (*n* = 8).

### 2.4 Biochemical studies

#### 2.4.1 Tissue preparation for biochemical analyses

Cortical tissue was homogenized in a Polytron homogenizer to obtain 10% (w/v) homogenate in 0.1 M potassium phosphate buffer (pH 7.4) containing 1 mM EDTA. The homogenate was centrifuged at 800 × *g* for 5 min at 4 °C to separate the nuclear debris. The supernatant was centrifuged again at 10,000 × *g* for 20 min at 4 °C to obtain post-mitochondrial supernatant (PMS), which was used for biochemical estimations.

#### 2.4.2 Acetylcholinesterase (AChE) activity

AChE activity was measured colorimetrically (Ellman et al., 1961) by spectrophotometric method and calculated as nmoles of acetylthiocholine iodide hydrolyzed/min/mg protein using a molar extinction coefficient of 1.36 × 10^4^ M^−1^ cm^−1^. OD was measured at 412 nm.

#### 2.4.3 Monoamine oxidase (MAO) activity

MAO activity was measured colorimetrically (Holt et al., 1997) and its activity calculated as μmoles of benzylamine hydrochloride (BAHC) hydrolyzed/min/mg protein using the molar extinction coefficient of 7.6925 M^−1^ cm^−1^. OD was measured at 280 nm.

#### 2.4.4 Nitric oxide (NO)

NO was assessed by quantifying the nitrite content in the cerebral cortex colorimetrically with the Griess reagent method (Grisham et al., 1996). The activity was calculated as μmoles of nitrite/mg/wet tissue by using the molar extinction coefficient of 4.0 M^−1^ cm^−1^. The OD of the reaction product was measured at 540 nm, and values were derived from a standard curve of sodium nitrite.

#### 2.4.5 Lipid peroxidation (LPO)

LPO was quantified spectrophotometrically (Mihara and Uchiyama, 1978). OD of the reaction supernatant was measured at 535 nm, and values are expressed as μmoles of thiobarbituric acid reactive substances (TBARS) formed/h/g protein using a molar extinction coefficient of 1.56 × 10^−5^ M^−1^cm^−1^.

### 2.5 Immunoblotting

#### 2.5.1 Tissue processing

The brain was immediately removed after the anesthesia with sodium pentobarbital, and the cerebral cortex was dissected carefully. Tissues were frozen on dry ice and stored directly at −80 °C until the protein was analyzed. The cerebral cortex was homogenized in RIPA lysis buffer (#R0278, Sigma Chemical Co.) with protease inhibitor cocktail (#I3786, Sigma Chemical Co.) in a Potter-Elvehjem homogenizer on ice at approximately 1,000× *g* for 5 min. Completely homogenized samples were centrifuged at 10,000× *g* using a refrigerated centrifuge for 10 min at 4 °C, and half the volume of supernatant was stored at −80 °C, and the rest of the supernatant was centrifuged at 100,000 × *g* at 4 °C for 1 h using Optima MAX-XP Ultracentrifuge (Beckman Coulter, Brea, CA, United States) to obtain the membrane fraction. Protein was estimated using Bradford Reagent (Bio-Rad, Hercules, CA, United States) at an absorbance of 595 nm in TECAN Infinite M-200 Microplate Reader using compatible *Magellan*™ data analysis software *version 6.6*.0.1(Tecan Group Ltd., Männedorf, Switzerland). Equal amounts of protein (30–40 μg) and Laemmli loading buffer (sample was kept at 37 °C only to avoid forming protein aggregates in gel) were electrophoresed under the same experimental conditions using 10% sodium dodecyl sulphate-polyacrylamide gel electrophoresis (SDS-PAGE) (Towbin et al., 1979). Gel with 1× SDS running buffer and pre-stained protein marker (Puregene 3 Color Prestain Protein Ladder, Genetix, India) was used for reference bands using Mini-PROTEAN^®^ Tetra Cell SDS-PAGE gel running unit (Bio-Rad). Protein was transferred from gel to polyvinylidene difluoride (PVDF) membrane (*mdi* Membrane Technologies, Ambala, India) and then was blocked in 5% (*w*/*v*) skimmed milk in TBST for 1 h before incubating with primary and HRP-conjugated IgG secondary antibodies (Supplementary Material, Supplementary Table S1). Proteins were detected with Clarity Western ECL substrate (Bio-Rad) in LAS-4000 (Fujifilm, Düsseldorf, Germany). The ratio of densities of protein bands of target proteins (GLT-1 and xCT) was normalized by protein density of β-actin using values provided by the Multi Gauge Image reader software (Fujifilm). The densitometric analysis was done by taking the average of all the replicates in the control group and later dividing it by the densities of bands from the replicates of the control, and two doses of BPA.

### 2.6 Quantitative reverse transcriptase polymerase chain reaction (qRT-PCR)

Total RNA was isolated from cortical tissue using the Trizol method by Tri Reagent (Sigma Chemical Co.). RNA concentration was determined by NanoDrop 1,000 version 3.7 (Thermo Scientific, Wilmington, DE, United States). Using 2 μg of total RNA, cDNA was synthesized from each tissue sample using a cDNA synthesis kit (Roche Molecular Systems, Inc., Pleasanton, CA, United States). The cDNA was then used as a template for individual PCR reactions using specific primer sets (Supplementary Material, Supplementary Table S2). Mouse-specific primers were designed by the Primer3 program, written by the Whitehead Institute. PCR reactions and their quantitative analysis were carried out using Light Cycler 480 SYBR^®^ Green PCR Kit and Light Cycler 480™ RT-PCR instrument and compatible software (Roche). Reactions were performed in triplicate, and the expression of each transcript was quantified by the relative standard curve method and normalized to that of β-actin. Fold change was calculated using the -ΔΔC_T_ method, and relative expression was represented (Livak and Schmittgen, 2001; Khandelwal et al., 2022).

### 2.7 Histopathological studies

Animals were perfused transcardially with ice-cold phosphate-buffered saline (PBS 0.1 M pH 7.4). Whole intact brains from three animals were carefully excised and fixed in the 4% paraformaldehyde solution for 48 h, and later embedded with paraffin. Coronal sections of 7 μm thickness were dewaxed and stained with hematoxylin and eosin (H&E) (Tabassum et al., 2017). Images were captured with the help of the microscope (Nikon Eclipse 80*i* microscope, Nikon Corporation, Tokyo, Japan) using a ×20 objective.

### 2.8 Statistics

Results were expressed as mean ± SEM. One-way analysis of variance (ANOVA) with Tukey’s *post hoc* test was used to analyze differences between the groups. The *p* < 0.05 was considered statistically significant. The trial version of GraphPad Prism eight software for Windows (GraphPad Software, San Diego, CA, United States) was used for the analysis of data.

## 3 Results

### 3.1 Behavior study

#### 3.1.1 Effect of BPA on learning and memory

**FIGURE 2:**
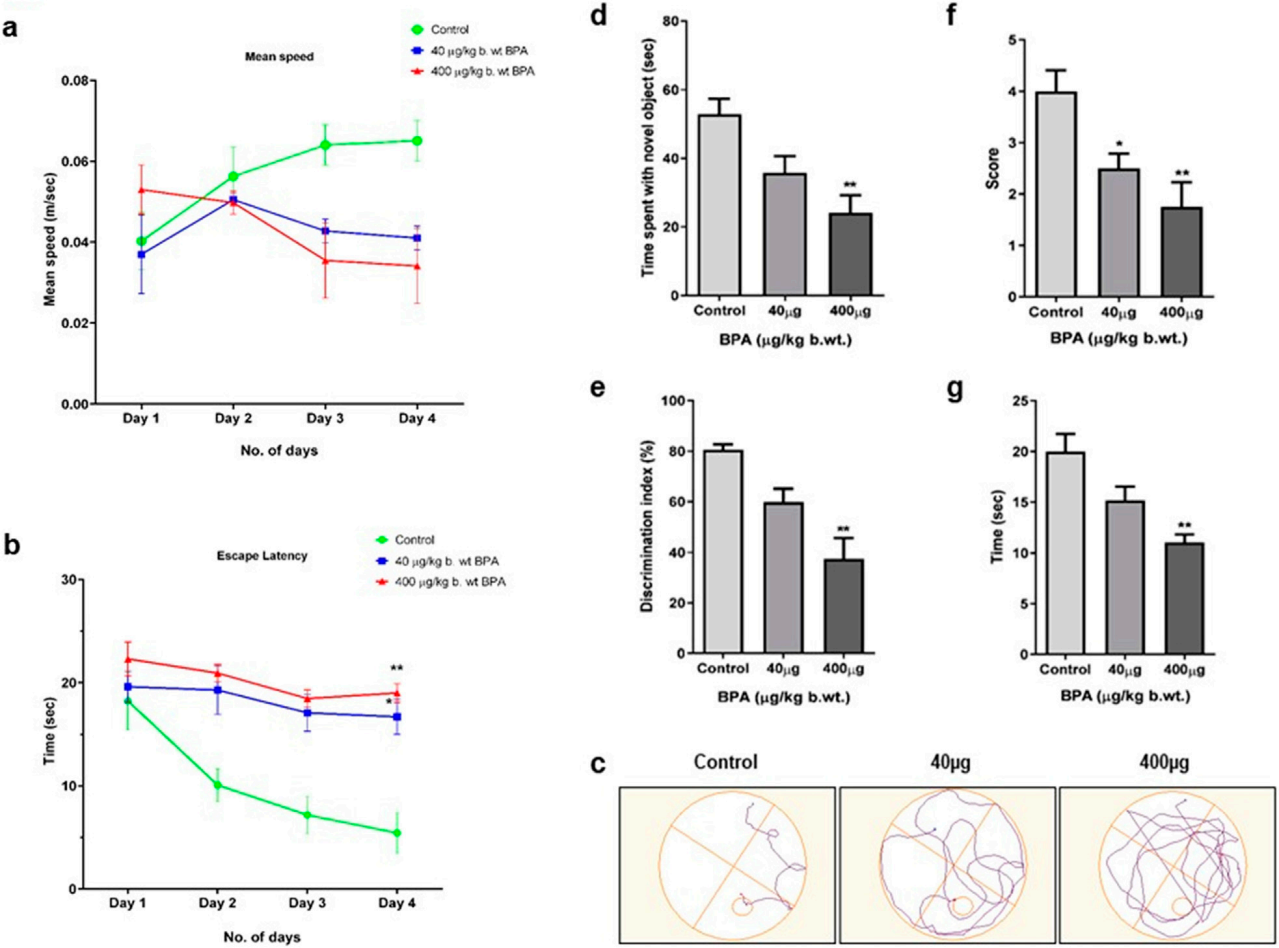
The effects of sub--chronic exposure the 40 μg to BPA on **(a–c)** Morris water maze (MWM) test for spatial learning, **(d,e)** Novel object recognition (NOR) test for non--spatial memory, **(f)** Grip strength by string test, and **(g)** rotarod test for sensorimotor coordination in male C57BL/6J mice. No significant change was observed 40 μg in the **(a)** mean speed in both the 40μg and 400 μg BPA. However, a significant decrease in **(b)** escape latency (*p < 0.05 and **p < 0.01) was observed in the 40 μg and 400 μg BPA treatment groups. **(c)** Path diagram of a mouse in a circular pool. **(d)** Recognition memory assessed by Novel Object Recognition test showed significantly (**p < 0.01) reduced **(d)** time spent with novel object and **(e)** Discrimination index in 400 μg BPA treatment, but 40 μg BPA showed no significant change. **(f)** Grip strength was significantly (*p < 0.05 and **p < 0.01) reduced, represented by the score in the string test in both 40 and 400 μg BPA treatment. **(g)** Rotarod test showed significantly (**p < 0.01) reduced time spent on rotating rod in 400 μg BPA treatment. No change was observed in 40 μg BPA treatment. All the data were analyzed by comparing with the control group animals. One--way ANOVA followed by a Tukey’s *post hoc* test was used to analyze the data. Data are expressed as the means ± SEM (*n* = 8).

As shown in Figure 2a, the mean swimming speed of animals in both the BPA-treated groups (40 and 400 μg/kg) was slower than the control group. However, the effect of BPA was not statistically significant on this parameter. Escape latencies (Figure 2b) of the BPA-treated animals were observed to be significantly longer (*p* < 0.05 and *p* < 0.01) than control group animals in the probe trial. Figure 2c shows the path diagram of the mice travelled in the circular pool. Data suggested that BPA-treated mice had reduced learning in the MWM test compared with the control group animals. Overall, our results indicated that BPA caused hippocampal and cortical damage, which was clearly represented by poor performance of exposed animals in spatial learning and memory assessment tasks.

#### 3.1.2 Effects of BPA on novel object recognition

BPA caused altered recognition memory in mice. Figure 2d shows the time spent by control and BPA-treated mice in exploration of the novel object in the assessment of recognition memory. Significantly decreased (*p* < 0.01) exploration time in the 400 μg BPA treatment group was observed compared with the control group mice. However, no significant difference was observed between the low-dose (40 μg) BPA-exposed and control group animals. BPA-treated mice showed a significantly (*p* < 0.01) reduced discrimination index in the 400 μg BPA treatment group, depicting an impaired ability of experimental animals to differentiate between novel and familiar objects when compared to the control group mice (Figure 2e). These results suggested that sub-chronic BPA exposure caused hippocampus-dependent impairment in short-term recognition memory. No significant change in the discrimination index was observed between the 40 μg BPA and the control group animals.

#### 3.1.3 Effects of BPA on grip strength and sensorimotor coordination

Muscular strength was assessed by grip strength scoring. As shown in Figure 2f, the mean grip score was significantly (*p <* 0.05 and *p <* 0.01) lowered in both BPA-treated groups compared to the control group mice. Sensorimotor coordination skill assessed by the rotarod- apparatus is shown in Figure 2g. We observed decreased sensorimotor coordination skills, shown by the lesser time spent on the rotating rod by BPA-treated as compared to the control group mice. Animals in the 400 μg BPA group withstand significantly (*p* < 0.01) less on the rotating rod due to altered motor coordination. However, despite poor performance, no significant score difference was observed in the 40 μg BPA-treated group compared to the control group animals. These results indicate that sub-chronic BPA exposure at a higher dose alters the sensorimotor coordination abilities of mice.

### 3.2 Biochemical studies

#### 3.2.1 Effect of BPA exposure on cerebral cortex AChE activity

**FIGURE 3:**
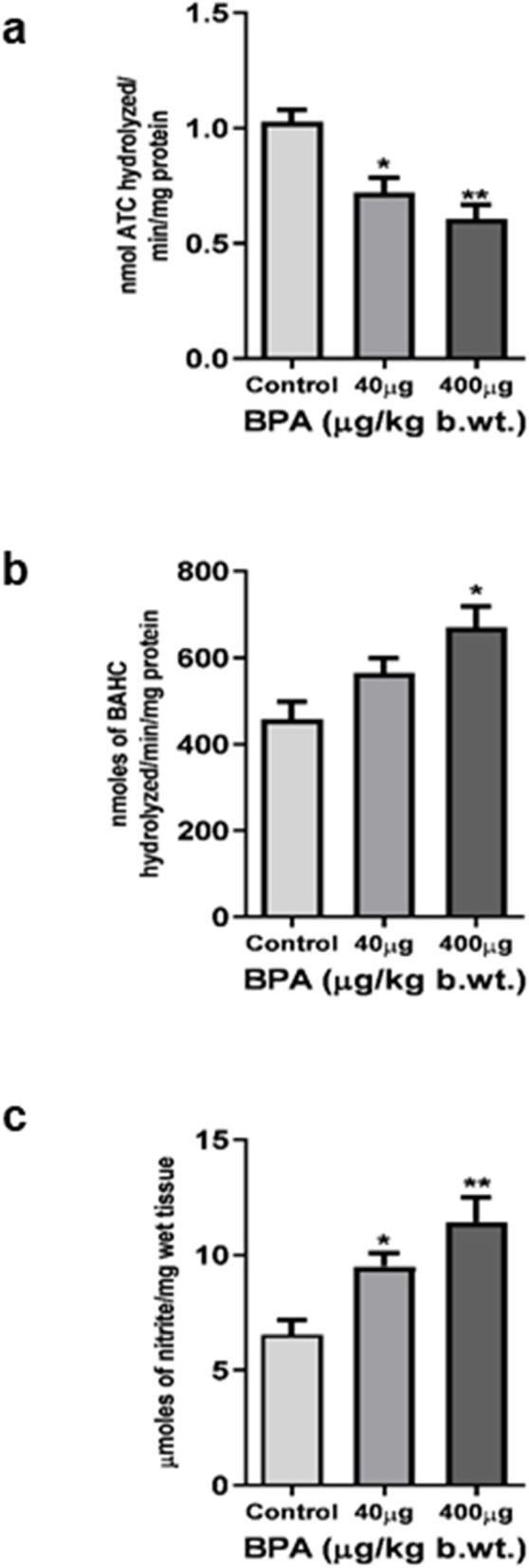
Enzymatic neurochemical markers of toxicity in the cerebral cortex of C57BL6/J male mice after the sub--chronic exposure to BPA. **(a)** Acetylcholinesterase activity was significantly (*p < 0.05 and **p < 0.01) reduced in both 40 and 400 μg BPA treatment groups, respectively. **(b)** Monoamine oxidase and **(c)** Nitric oxide activity was found to be significantly (*p < 0.05 and **p < 0.01), increased in both 40 and 400 μg BPA treatment groups. No change in the MAO activity of 40 μg BPA treatment groups was observed. Data has been analyzed by comparing with the control group animals. One--way ANOVA followed by a Tukey’s *post hoc* test was used to analyze the data. Data were expressed as the means ± SEM (*n* = 5).

The effect of BPA exposure on AChE activity in the cerebral cortex was shown in Figure 3a. BPA caused a significant decrease in AChE activity in 40 μg (*p* < 0.05) and 400 μg (*p* < 0.01) BPA treatment groups compared to the control.

#### 3.2.2 Effect of BPA exposure on cerebral cortex MAO activity

MAO activity in the mouse cerebral cortex is presented in Figure 3b. One-way ANOVA with Tukey’s *post hoc* test revealed that sub-chronic BPA exposure caused a significant (*p* < 0.05) increase in MAO activity in the 400 μg BPA treatment group in comparison to the control group animals. However, 40 μg BPA caused a significant change when data was compared with controls.

#### 3.2.3 Effect of BPA exposure on nitric oxide activity in cerebral cortex

The result of nitrite content as a measure of reactive nitrogen species load in the cerebral cortex was shown in Figure 3c. BPA exposure significantly (*p* < 0.05 and *p* < 0.01) increased the nitrite content in BPA-treated mice, both in 40 μg and 400 μg treatment groups, when the result was compared with the control group.

#### 3.2.4 Effect of BPA exposure on lipid peroxidation in cerebral cortex

**FIGURE 4:**
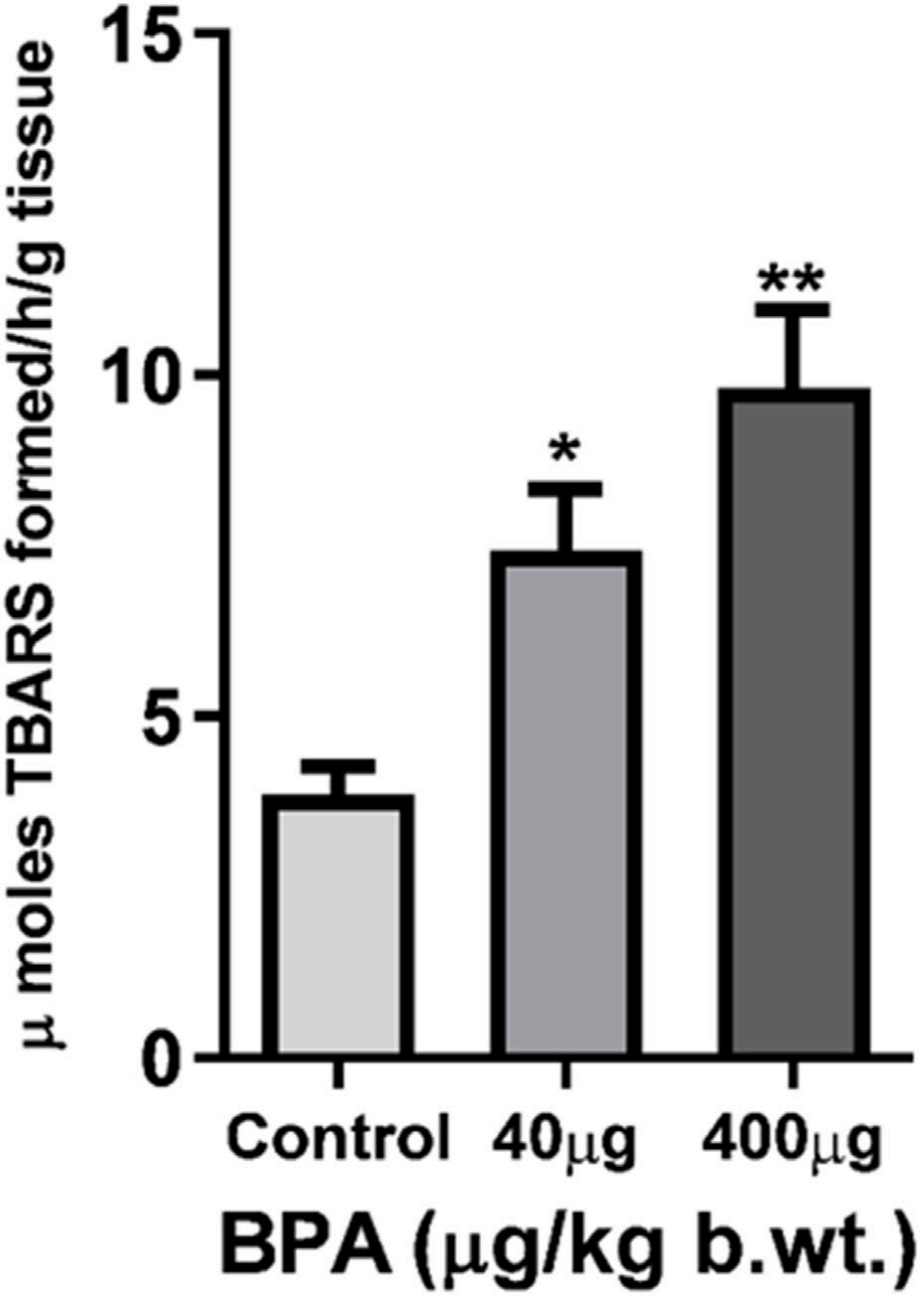
The effects of sub--chronic exposure to BPA on lipid peroxidation (LPO) in the cerebral cortex of C57BL6/J male mice. Significantly (*p < 0.05 and **p < 0.01) increased the TBARS level showed in both 40 and 400 μg BPA treatment groups. Data were analyzed by comparing with the control group animals. One--way ANOVA followed by a Tukey’s *post hoc* test was used to analyze the data. Data are expressed as the means ± SEM (*n* = 5).

TBARS levels were measured to determine the LPO in the cerebral cortex (Figure 4). BPA exposure significantly increased TBARS in the cerebral cortex of 40 μg (*p* < 0.05) as well as 400 μg (*p* < 0.01) BPA-treatment groups as compared to the control group.

### 3.3 Quantitative RT-PCR

#### 3.3.1 Effects of BPA on the mRNA levels of glutamate transporter/antiporter in the cerebral cortex

**FIGURE 5:**
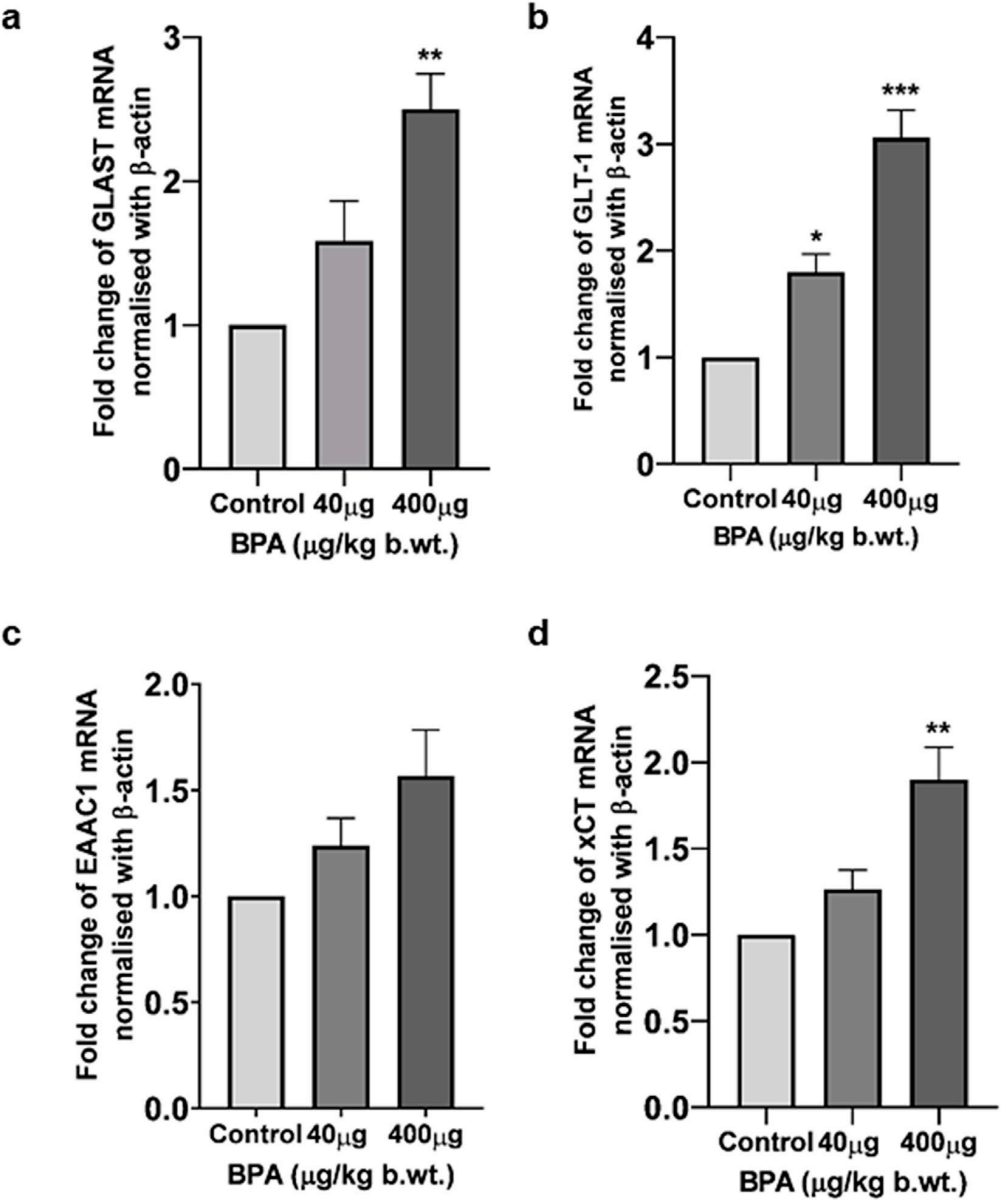
The effects of sub-chronic exposure to BPA on mRNA levels of glutamate transporters and antiporters in the cerebral cortex of mice by RT-PCR **(a-d)**. Significantly (*p < 0.05, **p < 0.01, and **p < 0.01) increased mRNA levels of **(a)** *Glast*, **(b)** *Glt-1, and* **(d)** *xCT* in 40 and 400 μg BPA treatment was observed, respectively, in the cerebral cortex in male C57BL/6J mice. Except for *Glt-1* mRNA levels, no change was observed in 40 μg BPA treatment in **(a)** *Glast*, **(c)** *Eaac1*, and **(d)** *xCT* mRNA levels. Data were analyzed by comparing with the control group animals. One-way ANOVA followed by a Tukey’s *post hoc* test was used to analyze the data. Data are expressed as the means ± SEM (*n* = 5).

The effect of sub-chronic BPA exposure on the gene expression (at mRNA level) of *Glast*, *Glt-1*, *Eaac1,* and *xCT* was presented in Figure 5. One-way ANOVA with Tukey’s *post hoc* test revealed a significantly (*p* < 0.01 and *p* < 0.001) increased mRNA expression of *Glast* (Figure 5a), *xCT* (Figure 5d), and *Glt-1* (Figure 5b) in the cerebral cortex of the 400 μg BPA group compared to the control group. In the 40 μg BPA group, the mRNA expression of only *Glt-1* (Figure 5b) was found to be increased (*p* < 0.05), and no significant change was found in either *Glast* (Figure 5a) or *xCT* (Figure 5d) expression. No statistically significant difference was observed in *Eaac1* (Figure 5c) mRNA levels in any of the BPA treatment groups when data were compared with the control group.

### 3.4 Immunoblotting

#### 3.4.1 Effects of BPA on the protein expression of glutamate transporter/antiporter in the cerebral cortex

**FIGURE 6:**
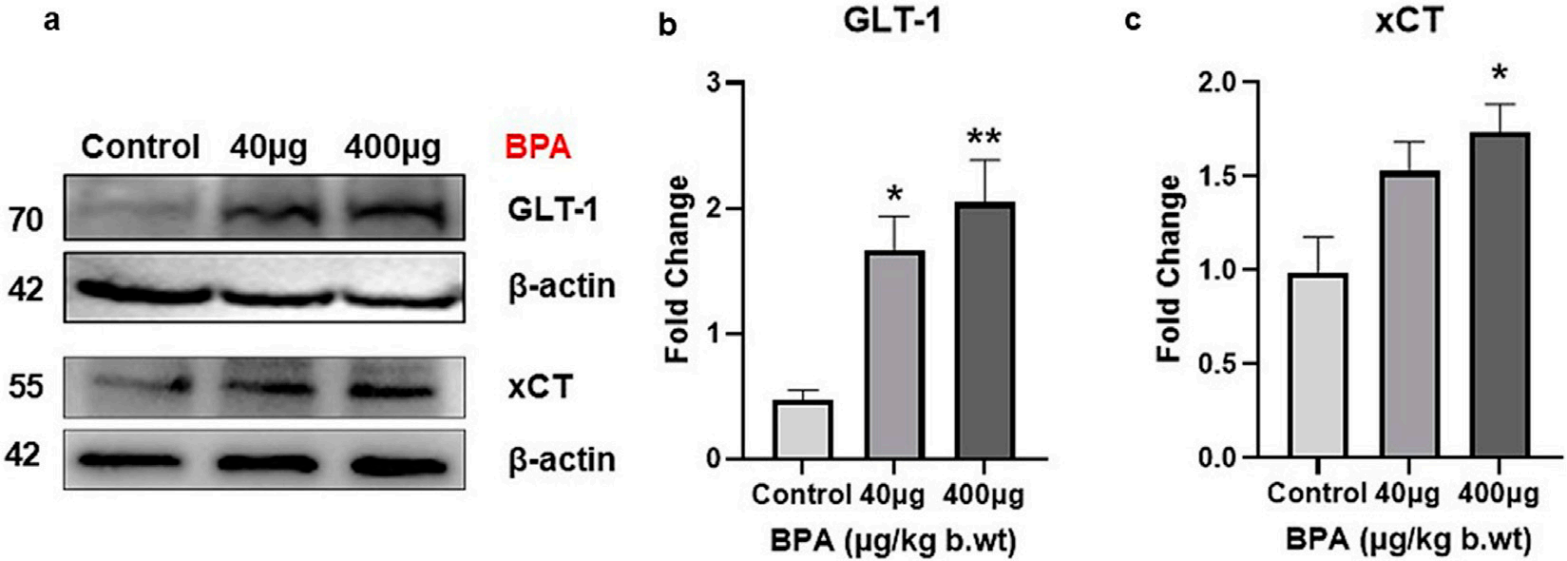
**(a)** Protein blots of Glu transporter (GLT-1) and antiporter (xCT) proteins in the cerebral cortex of mice. Densitometry analysis of Glu transporter **(b)** Glt-1 and antiporter **(c)** xCT protein expression in the cerebral cortex of C57BL/6J male mice on sub--chronic exposure to BPA. Significantly (*p < 0.05 and **p < 0.01) increased protein expression was observed in 40 and 400 μg BPA treatment groups. All the data were analyzed by comparing with the control group animals. One-way ANOVA followed by a Tukey’s *post hoc* test was used to analyze the data. Data are expressed as the means ± SEM (*n* = 5).

The effect of BPA exposure on the protein levels of GLT-1 and xCT in the cerebral cortex was presented in Figures 6a,b. One-way ANOVA with Tukey’s *post hoc* test revealed a significant increase (*p* < 0.01 and *p* < 0.05, respectively) in their levels in the BPA treatment compared with the control group. Increased xCT expression in the 40 µg BPA dose group was not significant compared with the control group.

### 3.5 Effects of BPA on the brain histology in the cortical and hippocampal tissue

**FIGURE 7:**
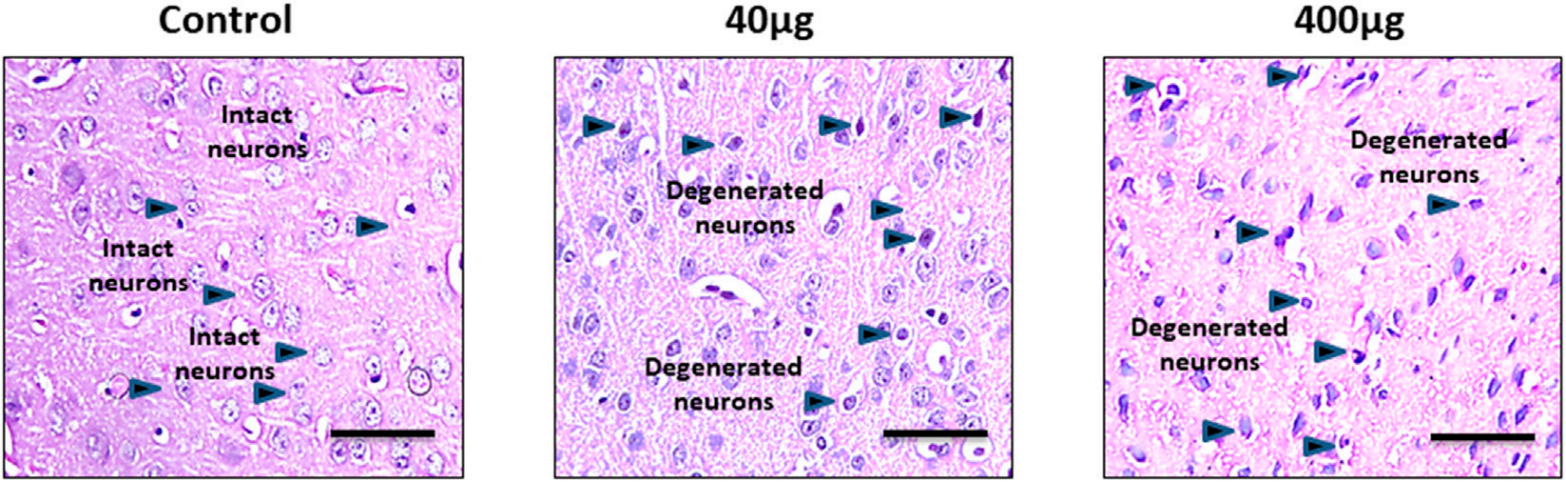
The effects of sub-chronic exposure to BPA on histopathology in the cerebral cortex of C57BL6/J male mice. H&E staining in the representative pictographs shows the dose-dependent histological alterations in the cortical tissue of mice. Arrows in 40 and 400 μg BPA treatment groups show the darkly stained degenerated neurons. Arrows in control sections show the intact neurons in normal tissue. Magnification = ×20 and scale is 50 μm.

**FIGURE 8:**
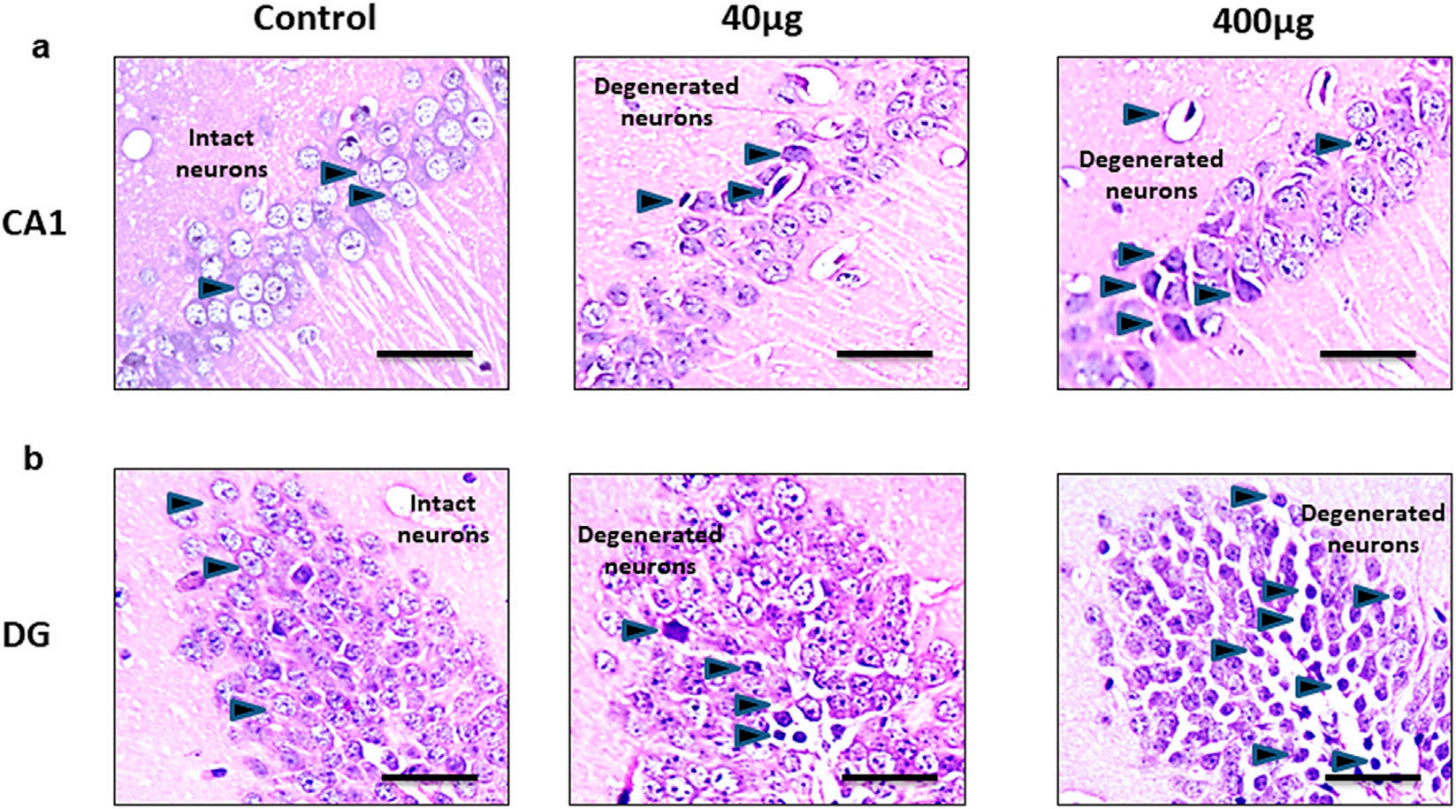
The effects of sub-chronic exposure to BPA on histopathology in the hippocampal regions of C57BL6/J male mice. H&E staining in the representative pictographs shows the dose-dependent histological alterations in the **(a)** Cornu Ammonis (CA1) and **(b)** Dentate gyrus (DG) region of mice. Arrows in 40 and 400 μg BPA treatment groups darkly stained sections representing degenerated neurons. Arrows in control sections show the intact neurons in normal tissue. Magnification = ×20 and scale is 50 μm.

Histopathological changes analyzed by - H&E staining in the cerebral cortex in Figures 7, 8 shows changes in the histoarchitecture of cortical sections from different groups of animals. Plenty of hyperchromatic cells representing cellular and spongiform degeneration in cortical tissue were observed in BPA-treated animals. There was pyknosis of nuclei (darkly stained punctuate nuclei) in the 40 μg and 400 μg BPA treatment groups (shown with the arrows). Additionally, BPA-induced hippocampal changes in the cornu ammonis 1 (CA1) and dentate gyrus (DG) regions in both the BPA treatment groups (Figures 8a,b). The control group showed a greater number of normal appearing cells with no pathological changes. An increased number of pyknotic nuclei (hyperpigmented, distorted nuclei) was observed in BPA-treated groups, with more pronounced changes in the high-dose group. Both the BPA-treatment groups showed hippocampal damage in CA1 and DG, with increased neuronal loss and vacuolated spaces in a dose-dependent manner. Changes such as neuronal loss, vacuolated spaces, and pyknotic nuclei represent cell death in BPA treatment groups (Supplementary Material, Supplementary Figures S2, 3). Supported by earlier studies, our results from histopathological analysis in the cerebral cortex and downstream hippocampal damage through cortico-hippocampal circuitry are accompanied by altered behavioral responses. Findings from BPA-induced biochemical disturbances in the cerebral cortex accompanying hippocampal histopathological changes are in agreement with *in vivo* models in which dysfunction in the cortex significantly contributes to hippocampal damage.

## 4 Discussion

Industrial chemicals, which have been reported to be safe for humans and other organisms, can potentially affect human health. One such renowned chemical pollutant is BPA (Biswas et al., 1987). In modern times, no chemical other than BPA has generated raging and heated debate among researchers, epidemiologists, policymakers, and activists, and there are ever-changing paradigms concerning exposure and effect (Wassenaar et al., 2017). Due to its broad-spectrum application in daily life, BPA has attained a ‘ubiquitous’ status. The sources of BPA include chemical industry discharge, plastic and electronic waste, medical waste, food, and food-contacting materials (Baluka and Rumbeiha, 2016; Naveira et al., 2021). The National Toxicology Program (NTP) of the United States stated that BPA is capable of negatively affecting the brain and behavior in fetuses, infants, and children (Hengstler et al., 2011). Evidence has been accumulating and demonstrating that gestational and adult exposure to BPA is associated with hyperactivity, aggression, anxiety, depression, and neurodevelopmental disorders (Inadera, 2015). Most of the previously published reports focused on either gestational or prenatal exposure-mediated effects of BPA on brain and behavior. Very few studies have explored the mechanistic underpinnings of adult exposure to BPA and associated neurotoxicity. Thus, in the current study, we are focusing on the effect of BPA exposure in the cerebral cortex, which controls multiple high-level functions of the brain.

BPA is reported to alter dopamine-dependent neurotransmission, resulting in hypersensitivity of drug-induced hyperlocomotion, impaired working, and recognition memory (Tian et al., 2010; Zhang et al., 2019). Perinatal maternal exposure to BPA alters spatial and passive avoidance memory in juvenile and adult rodents (Wu et al., 2020; Xu et al., 2010). We observed that sub-chronic BPA exposure induced impaired behavioral responses in MWM tasks. We found decreased escape latency (Figure 3a) and mean swimming speed (Figure 3b) of mice to reach the safe platform in the target quadrant in the circular pool in BPA-exposed mice as compared to controls. BPA-exposed animals found it difficult to find the safe platform in the MWM tests (Figure 3c). This shows that BPA exposure severely altered spatial learning, which may be due to BPA-induced damage to both the hippocampus and the cortex, as both are involved in memory processing, which is shown by others as well (Preston and Eichenbaum, 2013). A NOR test was applied to assess the changes in the recognition memory after BPA exposure. The low dose of BPA at 40 µg showed no significant difference compared to the control group (Figure 3c). On the other hand, the higher dose (400 µg) group showed significantly altered recognition memory, shown by significantly reduced total time spent exploring novel objects (Figure 3c) and a discrimination index (Figure 2d). BPA-treated animals spent more time with the familiar object, representing altered short-term memory, as shown by their poor performance in the NOR test. These results are in agreement with the results from earlier published reports depicting memory and learning impairment after BPA exposure in MWM (Xu et al., 2010) and the Y-maze task (Khan et al., 2019; Poimenova et al., 2010). BPA is also reported to alter cognitive responses (Mahdavinia et al., 2019; Preciados et al., 2016), which in our study was represented by significantly reduced grip strength (Figure 3f) and sensorimotor coordination (Figure 3g) abilities, assessed by string-based grip strength and the rotarod test, respectively. However, no significant effect was found at the lower dose of BPA (40 µg). BPA-induced cognitive dysfunctions and motor defects are one of the major behavioral defects reported earlier (Hu et al., 2020; Tandon et al., 2020). We have further discussed the effect of BPA on grip strength and sensorimotor coordination in relation to neurochemical markers of toxicity in the latter part of this section. Behavioral changes are greatly influenced by neurotransmitters like Glu, and its receptors and transporters which play a mechanistic role in the cognitive and sensorimotor responses (Homayoun et al., 2004). Altered activity of neuro-specific biomarker enzymes such as MAOs, which regulate the level of catecholamines and serotonin to maintain optimum neurotransmission, and AChE, which regulates the activity of neurotransmitter acetylcholine, have been widely accepted metrics to measure the neurotoxicity of chemicals (Souza et al., 2020). Inhibition of AChE leads to over-stimulation and activity of acetylcholine in the brain, causing dysregulation of neuronal excitability, which induces synaptic plasticity and synaptic transmission (Souza et al., 2020). We found significantly reduced activity of AChE in BPA-treated groups (Figure 3a). Reduced AChE may have caused acetylcholine accumulation, leading to cholinergic synapses, causing overactivation of nicotinic and muscarinic receptors (Bjørling-Poulsen et al., 2008). A previously published report by Luo et al. (2013) also supports our results on BPA-induced reduced activity of AChE in brain tissue. On the other hand, BPA caused an increase in MAO activity in the cerebral cortex (Figure 3b). MAOs are involved in the metabolism of monoamines (Guo et al., 2014), reactive oxygen species (ROS) and reactive nitrogen species (RNS), which have been reported to cause neurodegeneration (Wang et al., 2019b).

Stimuli for ROS generation can be both exogenous and endogenous. Leaks in the electron transport chain in mitochondria mainly cause endogenous ROS production, especially O_2_^·−^. This O_2_^·−^ can react with NO^·^, an RNS, and generate potent oxidant radical peroxynitrite (ONOO^−^) (Redza-Dutordoir and Averill-Bates, 2016). Studies have shown that NO can mediate neurotoxicity leading to neuropathological disorders (Yao et al., 2004). Additionally, NO is involved in regulating synaptic transmission in the brain (Sajad et al., 2009). We have also found an increased nitrite content, signifying the elevated NO activity in the cerebral cortex of mice exposed to BPA (Figure 3c). Our results involving AChE, MAO, and NO strongly support our hypothesis that BPA exposure causes poor sensorimotor and grip strength performance. Reduced AChE activity causes altered regulation of acetylcholine at neuromuscular junctions, which affects sensorimotor and muscular performances (Costa and Cairrao, 2024). Simultaneously, BPA exposure caused significantly increased MAO activity, which proves that it increases the breakdown of monoamines, which is reported to diminish serotonergic and dopaminergic signaling, causing cognitive defects (Costa and Cairrao, 2024). NO exacerbates neurotoxicity by RNS generation, which caused poor sensorimotor and grip strength performance (Nazmy et al., 2025). RNS also works together with ROS-induced oxidative damage (Sajad et al., 2009; Wang et al., 2019b). BPA exposure causes hydroxy radical formation in the rat striatum, leading to oxidative stress-induced neurotoxicity, mitochondrial injury, decreased neurogenesis, increased neurodegeneration, and behavioral alterations in the hippocampus after the low-dose exposure in adult rats (Kabuto et al., 2004). Oxidative damage due to ROS leads to damage to lipid and protein functions in the brain (Khan et al., 2019). We observed that low-dose BPA exposure in adult animals caused an increase in LPO (Figure 6), which is considered A measurable biomarker of oxidative stress and related behavioral defects (Akram et al., 1987; Khan et al., 2018; Anjum et al., 2011). Glu homeostasis contributes significantly to regulating neurotoxicity affecting memory and sensorimotor responses. Elevated levels of extracellular Glu cause over-activation of Glu transporters, leading to excitotoxicity and neuronal death (Lau and Tymianski, 2010; Mehta et al., 2013). Glu dysregulation also contributes to neuropathology-mediated neurological disorders like multiple sclerosis, epilepsy, schizophrenia, and amyotrophic lateral sclerosis (McCullumsmith et al., 2016; O’Donovan et al., 2017; O’Donovan et al., 2017; Scott et al., 2002; Pampliega et al., 2011). These neurological disorders show aberrantly elevated expression of Glu transporters and antiporters as (McCullumsmith et al., 2016; O’Donovan et al., 2017; O’Donovan et al., 2017; Scott et al., 2002).

Several studies have reported the neurotoxic potential of BPA by altering Glu homeostasis directly and indirectly which involved the mechanism of endocrine disruption, oxidative/nitrosative stress, neurotransmitter disturbances, neuroinflammation, and cell death (Thayil et al., 2020; Khan et al., 2018; Wang et al., 2019a; Khan et al., 2019). BPA has been reported to alter Glu metabolism and its accumulation in cultured primary astrocytes (Zhang et al., 2025). BPA also triggered excitatory neurotoxicity in human embryonic stem cell-derived cortical neurons (Wang et al., 2019b). Glu transporters play a vital role in managing optimal Glu concentrations to avoid excitotoxicity (O’Donovan et al., 2017). Till now, BPA-induced neurotoxicity has been studied only in the context of Glu receptors and their effects on the neuroendocrine axis (Zhang et al., 2025). Though this strengthens the concept that BPA can alter normal Glu regulation, and there is a huge scope to study other aspects of Glu-induced neurotoxicity. Not much work has been done looking into BPA-induced changes in Glu transporters, which encouraged us to conduct this study. We assessed of the impact of exposure of BPA up to 60 days on adult male mice behavior, expression of EAATs (*Glast, Glt-1, and Eaac1*), and antiporter (*xCT*), in the mouse cerebral cortex. Mice were orally exposed to two distinct doses of BPA. Doses of BPA (40 μg and 400 μg) selected in this study were much less than the NOAEL of BPA (5 mg/kg/b.wt.) (EFSA Panel on Food Contact Materials, Enzymes, Flavourings and Processing Aids (CEF), 2015; U.S. Environmental Protection Agency, 2010). It has been reported that BPA has demyelinating and axonal degenerative effects in the mouse cerebral cortex at those doses (Khan et al., 2019; Tandon et al., 2020). We observed that doses of BPA used in our study are lower than environmentally representative doses of BPA (Lowest observed adverse effect level LOAEL)-50 mg/kg.b.wt per day for humans, (Wang et al., 2019a; Wang et al., 2019b) *via* the oral route can impart changes in the Glu-regulating genes, and their altered expression can cause adverse behavioral and neuropathological changes. We observed an increased mRNA level of *Glast*, *Glt-1, Eaac1,* and *xCT* in the cerebral cortices of BPA-treated animals (Figures 5a–d). Furthermore, we have found significantly upregulated GLT-1 and xCT protein expression in the cerebral cortex of male mice (Figures 6b,c, respectively). A low dose of 40 µg was unable to significantly change the mRNA levels of *Glast*, *Glt-1*, *Eaac1,* and *xCT* compared with the control group. Some other environmental toxicants, such as paraoxon and chlorpyrifos, are reported to cause aberrant changes in the levels of Glu transporters in the hippocampus and cerebral cortex of rodents (Slotkin et al., 2010; Zare et al., 2019). Similarly, arsenic is also reported to increase the expression of Glu transporters in rodents (Nelson-Mora et al., 2018; Ramos-Chávez et al., 2015). Studies have shown that BPA exposure negatively affects ATP levels through reduced Na^+^/K^+^ ATPase activity in the brain (Abdou et al., 2022). Reduced Na^+^/K^+^ ATPase activity in the brain has been reported to cause transporter reversal and Glu buildup in the extracellular space (Crino et al., 2002; Rossi et al., 2000). This provides strength to our results, showing the mechanism of BPA exposure causing overactivation of Glu transporters. Mechanistically, as stated earlier, overactivation causes transporter reversal, further leading to elevated Glu levels in the synaptic cleft (Rossi et al., 2000). A similar phenomenon with abnormally activated Glu transporters has been reported in pilocarpine-induced hippocampal and neocortical epilepsy, and post-mortem cortical tissue of epilepsy patients (Crino et al., 2002), and brain ischemic tissue. Glu build-up has been reported to cause neuronal hyperexcitability and episodes of seizures (Crino et al., 2002; Rossi et al., 2000). Other neuropathological conditions, such as endotoxin-induced oligodendroglial toxicity (Domercq et al., 1950), α-synucleinopathy (Diniz et al., 2020), Alzheimer’s Disease (Scott et al., 2002), multiple sclerosis (Evonuk et al., 2015), schizophrenia (O’Donovan et al., 2017), and cortical hypoxia (Roberts et al., 2014), also witness abnormally elevated xCT transporters. As explained in the beginning, transporter reversal-mediated elevated Glu into the extracellular space causes overactivation of NMDA receptor-mediated Ca^2+^ ion influx in the cell far beyond pathological levels, causing excitotoxicity-induced cell death (Rossi et al., 2000). We have also reported that BPA exposure causes significant neuronal cell death *in vitro* (Supplementary Material, Supplementary Figure S4). This explains that BPA exposure also follows a similar mechanism of neurotoxicity as reported by others. BPA-induced neurotoxicity involves disruption of crucial signaling pathways regulating autophagy, synaptic plasticity, learning, memory, neuronal survival, etc. (Santoro et al., 2019). NF-κB, a pro-inflammatory transcription factor, is found to be overactivated in the BPA-exposed animals, which shows its direct relationship with oxidative stress and neuroinflammation (Shih et al., 2015). Autophagy regulates inflammation through the Drp1 and p62 pathway, and BPA is reported to induce p62 accumulation in neuronal cells, suggesting its neurotoxic potential (Lee et al., 2021). Extracellular signal-regulated kinase (ERK), cyclic AMP response element-binding protein (CREB), and brain-derived neurotrophic factor (BDNF) signaling axis regulates neurotrophic signaling, and BPA is earlier reported to inhibit phosphorylation of these key proteins. Inhibition of these regulatory proteins causes altered cellular differentiation efficiency, reduced survival, and synaptic plasticity, ultimately causing memory-related defects (Wang et al., 2016).

BPA exposure in the rats has been reported to damage cortical tissue, showing cellular damage by histological markers (Aydoğan et al., 2008). In our study, the focus was on assessing BPA-induced biochemical, molecular, and histopathological changes based on earlier reports of cortical damage by BPA exposure through oxidative stress, glutamate dysregulation, and synaptic dysfunction (Khadrawy et al., 2016; Elsworth et al., 2015). Additionally, we have performed histopathological analysis in the hippocampus due to its vulnerability to structural and functional damage (Al-Shami et al., 2025; Alabi et al., 2023). Due to strong cortical-hippocampal connectivity, cortical damage by free radicals and inflammatory factors can influence hippocampal integrity, causing behavioral changes and neurodegeneration (Elsworth et al., 2015). In Figures 7, 8, H&E staining of the cortical and hippocampal regions of the control group brain tissue showed no morphological changes and normal histoarchitecture after 60 days of exposure to vehicle. Cortical and hippocampal regions, CA1 and DG, exhibited nuclear pyknosis and hyperchromatic cells representing spongiform degeneration. Damage to hippocampal tissue revealed downstream effects of biochemical dysregulation in the cerebral cortex. These results directly correlate with the cognitive and sensorimotor outcomes after BPA exposure mentioned earlier in this section. Our study is in conjunction with published reports, suggests that BPA exposure in adults produces significant neurotoxicity even at lower doses. BPA exposure aberrantly escalates Glu transporter expression in the cerebral cortex and has an active role in altering spatial learning and muscle function in adults.

## 5 Conclusion

Our results showed that the mRNA and protein expression of ionotropic Glu transporters (EAATs) and antiporter (xCT) increased in the cerebral cortex after the low-level sub-chronic exposure to BPA. BPA exposure may create a favorable environment for various neurological disorders in the exposed population. Also, our study explains the cognitive and neuromuscular damaging capability of low doses of BPA at sub-chronic exposure, represented by impaired spatial learning, recognition memory, and sensorimotor coordination abilities.

## 6 Limitations and future scope

All the molecular analyses were performed in the cortical tissue to address our cerebral cortex-focused hypothesis, while memory assessment tasks and histopathological analysis in hippocampal tissue provided supportive evidence of neurodegeneration. Analysis of the expression pattern of Glu transporters in hippocampal tissue, supporting our results after oral BPA exposure, is limited. There is still scope for further studies in a bigger sample size and inclusion of both sexes to further support the hypothesis of the current study and outcomes. Modulation of Glu transporters with appropriate activators of these protein targets could be an additional revelation that may support the hypothesis that BPA affects the Glu expression and related behavioral changes in the exposed mice. Also, as per the reviewer’s suggestion, quantifying protein tyrosine nitration could be a strengthening option for this manuscript, but it is limited in this study. The current study itself is critical and paves the way for further detailed investigation based on the role of Glu transporters in the BPA-mediated neurotoxicity of adult BPA exposure.

## Data Availability

The raw data supporting the conclusions of this article will be made available by the authors, without undue reservation.
