# Supplementary material for: Role of bisphenol A in the aberrant activation of ionotropic glutamate transporters in the cerebral cortex and altered behavioral responses in C57BL/6J mice
Source: Front Toxicol. 2026 Jan 12;7:1680589. doi: 10.3389/ftox.2025.1680589 (PMC12832418; doi:10.3389/ftox.2025.1680589)
Supplement: Supplementary file 1 [file Supplementaryfile1.docx]

**SUPPORTING MATERIAL FOR PUBLICATION**

**Table S1: List of primary and secondary antibodies utilized in the study.**

| **S.No.** | **Primary Antibody** | **Dilution** | **Manufacturer** | **Catalogue No.** |
| --- | --- | --- | --- | --- |
|  | GLT-1 | 1:1000 | abcam | ab41621 |
|  | xCT | 1:1000 | abcam | ab175186 |
|  | β-Actin | 1:1000 | Santa Cruz Biotechnology, USA | sc-47778 |
|  |  |  |  |  |
|  | **Secondary Antibody** | **Dilution** | **Manufacturer** | **Catalogue No.** |
| 1. | Goat-anti rabbit | 1:10000 | Santa Cruz Biotechnology, USA | sc-2030 |
| 2. | Goat-anti mouse | 1:10000 | Santa Cruz Biotechnology, USA | sc-2039 |

**Table S2: List of mouse-specific oligonucleotides utilized in the study with the maximum amplicon size of 200 base pairs.**

| **S.No.** | **Gene** | **Forward Primer** | **Reverse Primer** |
| --- | --- | --- | --- |
|  | *Glast* | TTGCAGCAAGGGGTCCGCAA | GCAGTGACCGTGAGCAGAACGA |
|  | *Eaac1* | GCCAGTTACATTCCGCTGTGCG | TGCAGACCATGACCCGAGAGCA |
|  | *Glt-1* | CAAGTCTGAGCTGGACACCA | GTGTGCGGCATAGACACACT |
|  | *xCT* | GATTCATGTCCACAAGCACAC | AGAGCATCACCATCGTCAGA |
|  | *β-actin* | AGACTTCGAGCAGGAGATGG | CAACGTCAC ACTTCATGATGG |

**Figure S1**

**
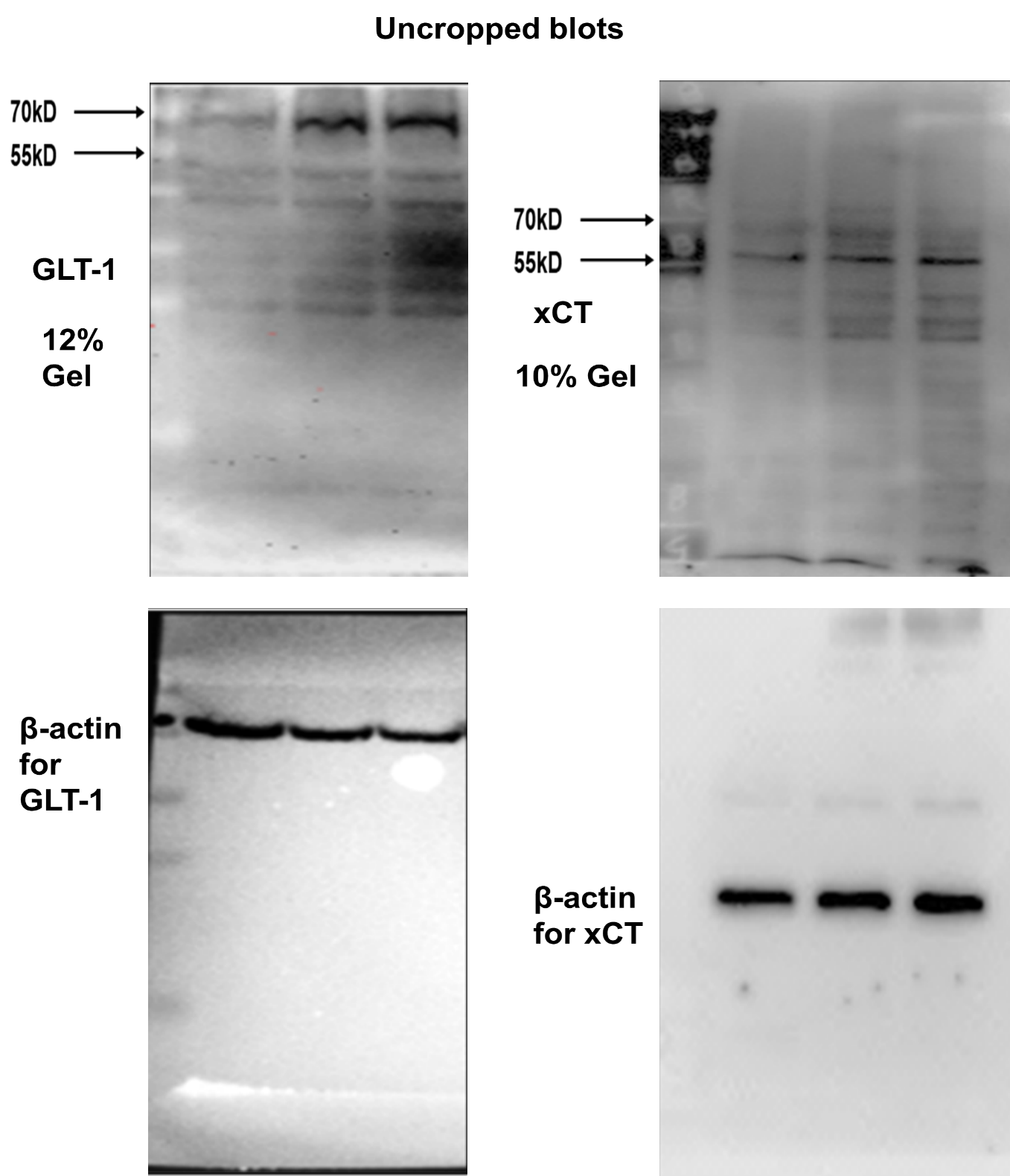
**

Full uncropped blots representing effects of BPA on the expression of GLT-1/EAAT2 and xCT with respective loading control proteins in cerebral cortex of male mice.

**Figure S2**

*
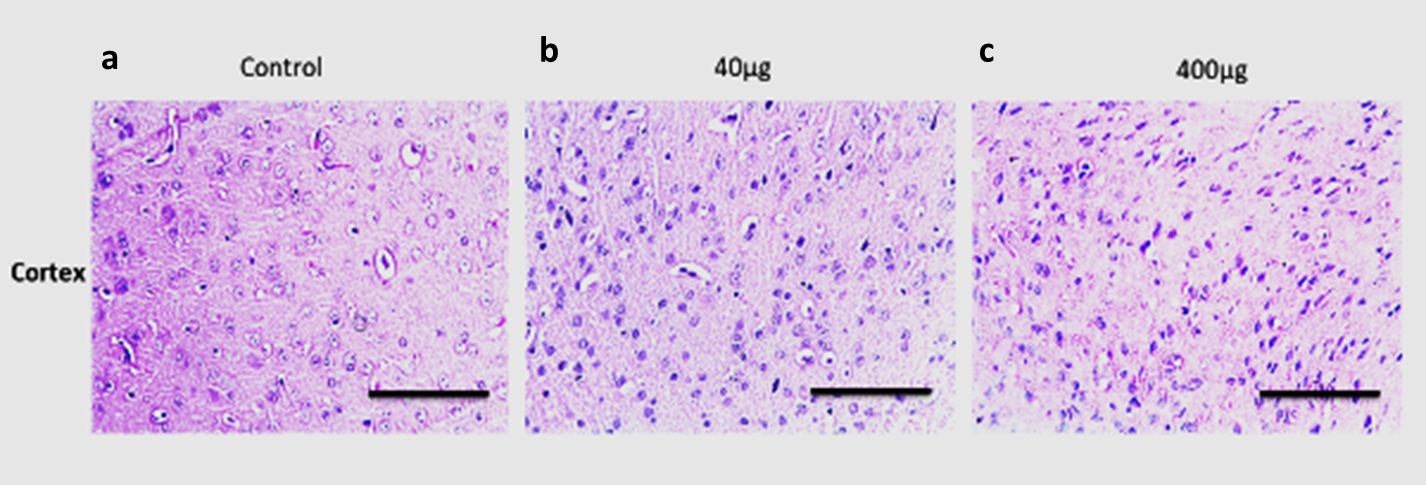
*

Figures representing effects of BPA on the cortical histoarchitecture in (a) control and (b-c) BPA treated in male mice.

**Figure S3**

*
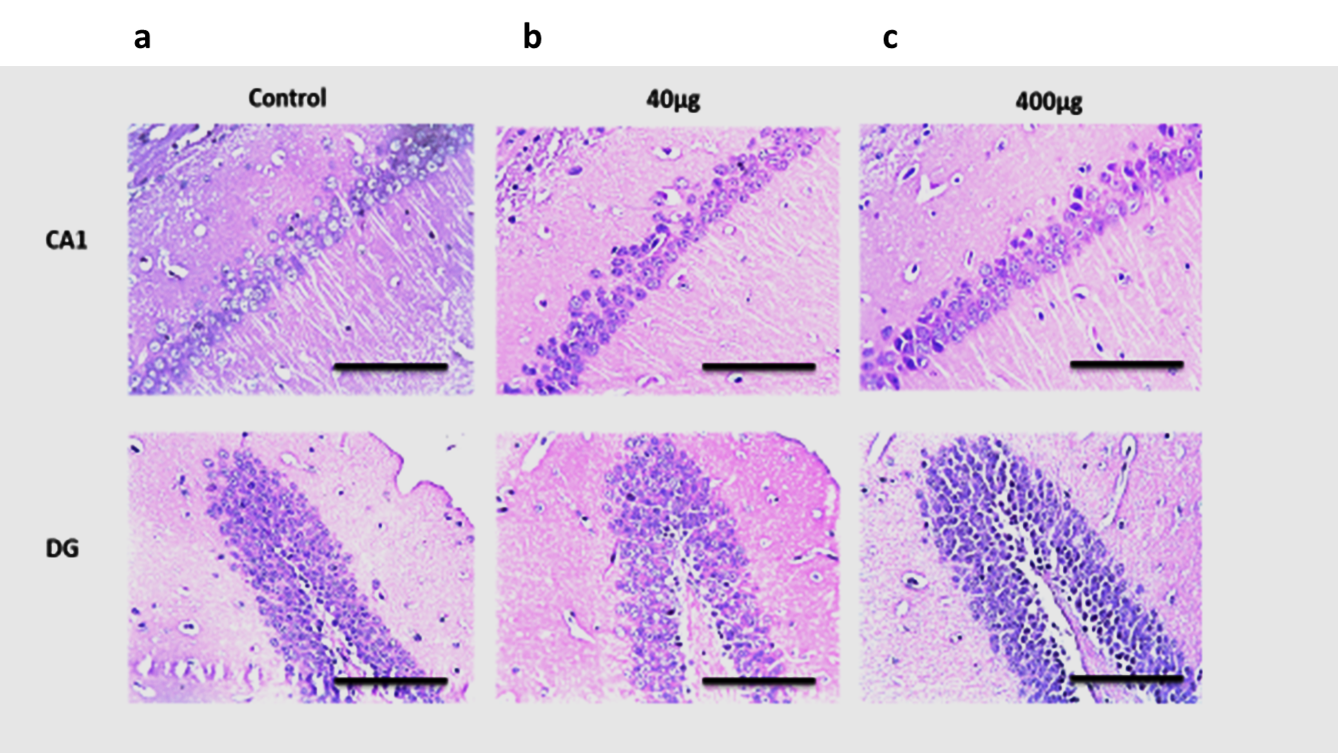
*

Figures representing effects of BPA on the cortical histoarchitecture in (a) control and (b-c) BPA treated in male mice.

**Figure S4**

*
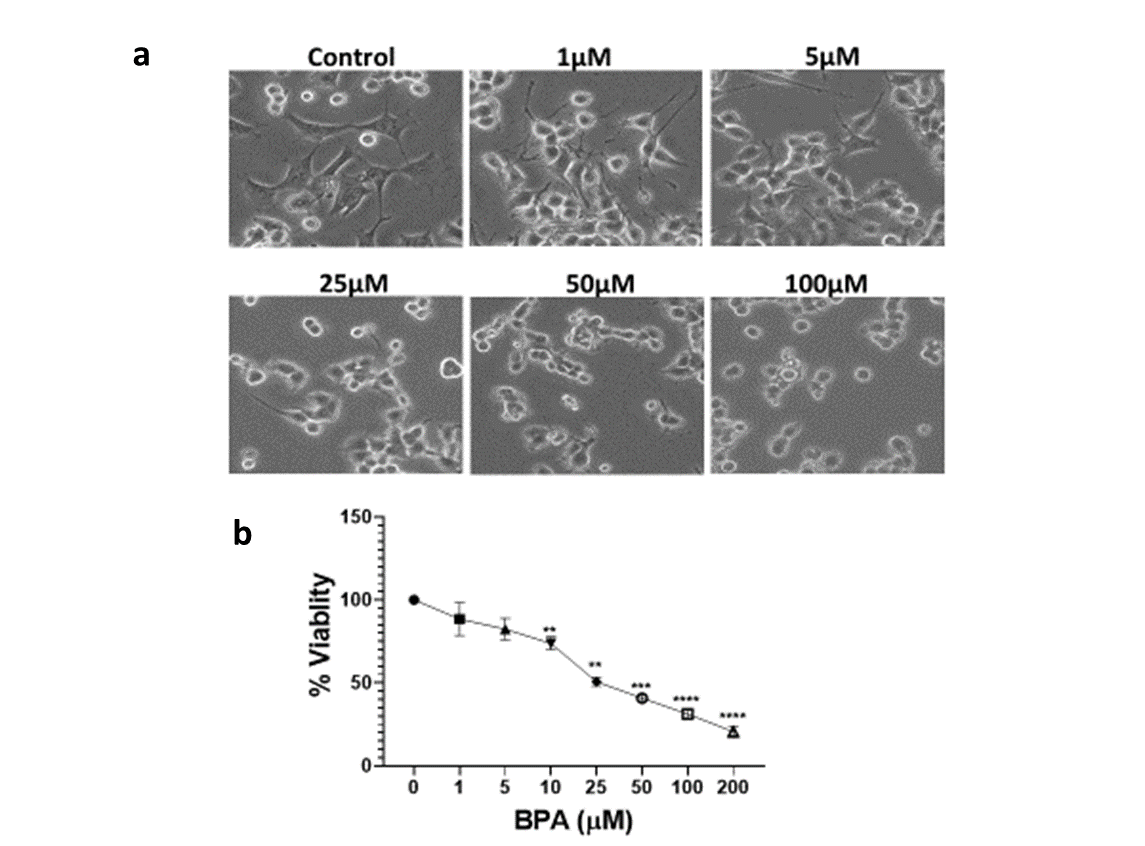
*

Effects of BPA exposure on Neuro-2a viability. a. Morphological changes in control and BPA (1µM-100 µM) treated cells for 24 hours. b. Percentage of surviving cells detected by the MTT assay after 24 h of incubation and shown in the graph. 10-200 µM BPA showed significantly reduced viability of Neuro2A cells, respectively. ***p*< 0.01, ****p*< 0.001, *****p*< 0.0001.
